# Increasing the psychosocial focus in child developmental assessments: a qualitative study

**DOI:** 10.1186/s12887-023-03849-x

**Published:** 2023-01-25

**Authors:** Sarah de Voss, Philip Wilson, Sofie Saxild, Gritt Overbeck

**Affiliations:** 1 Department of Public Health, Centre for General Practice, University of Copenhagen, Øster Farimagsgade 5, Opg. Q, CSS, Bg. 24, Postboks 2099, 1353 København K, Denmark; 2 Centre for Rural Health, Institute of Applied Health Sciences, University of Aberdeen, Old Perth Road Inverness, IV2 3JH Aberdeen, Scotland

**Keywords:** Psychosocial, Mental wellbeing, Mental health, Preventive care, Child, Children

## Abstract

**Background:**

Previous studies have indicated a need for increased psychosocial focus on children and their families to improve children’s wellbeing and mental health. Child developmental assessments could be a place to implement changes to achieve this. A standardised record might be helpful to clinicians trying to increase psychosocial focus. The aim of this study is to investigate clinical barriers and facilitators when introducing standardised child records with increased focus on psychosocial wellbeing and mental health into child developmental assessments.

**Methods:**

This is a qualitative study based on 12 semi-structured interviews with four midwives and nine doctors who carry out child developmental assessments in general practice. Data is analysed in the framework of Normalisation Process Theory.

**Results:**

General practice-based clinicians were positive towards increasing the psychosocial focus in child developmental assessments. The main barriers when clinicians used the standardised child records were: feeling forced to ask certain questions, in turn making the conversation rigid; leaving less room for parents to bring up other issues; making clinicians feel awkward when addressing problems that they cannot solve; the need for extended consultation time; and medico-legal concerns when registering findings. The experience of positive aspects when using the standardised child records facilitated continuous use of the records. Positive aspects included having a standardised approach to recording important findings, thereby uncovering psychosocial problems that could potentially be overlooked. Additionally, structured observation of parent–child interaction and gaining a new vocabulary to describe the findings were valued by clinicians. Balancing a standardised approach with clinicians’ ability to steer the consultation and explore topics in depth while preserving the potential for patients to bring up other issues became an important theme.

**Conclusion:**

Clinicians need to be well-equipped to handle psychosocial problems through coping strategies, referral options and communication techniques in the psychosocial domain. The parent–child-interaction assessment might expose potentially dysfunctional parenting behaviours and could improve communication between health professionals. Implementing standardised child development records with an increased psychosocial focus is feasible but improvements could optimise the use of the records. Parental views on an increased psychosocial focus during child developmental assessments should be investigated prior to further implementation.

**Trial registration:**

Trial registry number for the FamilieTrivsel (Family Wellbeing) trial: NCT04129359.

**Supplementary Information:**

The online version contains supplementary material available at 10.1186/s12887-023-03849-x.

## Background

There is a strong association between children’s early mental development and problems later in life – including learning disability, educational failure, criminality, as well as a range of physical and mental illness [1, 2]. Development is affected by numerous factors within the family, including parental mental health and wellbeing [3–5], childhood adversity experienced by the parents themselves [6, 7], parent–child interaction [8, 9] and the security of the child’s attachment to the parents [10, 11]. Regular assessment of the child’s psychosocial environment may therefore be beneficial because it might detect potentially remediable factors that may adversely affect child health outcomes [12, 13].

Early detection of concerns and targeted interventions can support child development, minimise cognitive or physical impairment in children [14–17] and provide cost-effective benefits to national economies [18]. Detection of risk factors is, however, necessary to identify children in need of targeted interventions [14–17]. In most developed countries preventive child health examinations (‘developmental assessments’) follow children’s health and development and provide a space for parents to bring up concerns during the early years of childhood [19]. This approach to child health surveillance is not considered a screening programme as few if any of the constituent activities fulfil the widely accepted WHO screening criteria [19, 20]. There are international variations in where developmental assessments are conducted (general practice, special clinics or hospital) and by which professionals (general practitioners (GPs), paediatricians, midwives, nurses). National guidelines for developmental assessments often provide detailed descriptions of the physical examinations and developmental milestones but generally fail to include specific advice on how to perform psychosocial or developmental assessments [19, 21, 22]. Psychosocial aspects are treated in an unstandardised, inconsistent matter in developmental assessments and often downgraded or completely omitted in favour of the physical examination [23–25]. An increased focus on psychosocial and mental wellbeing in existing developmental assessments has received support from several sources including the World Health Organization [23, 26, 27]. To achieve increased focus on psychosocial and mental wellbeing, a standardised, consistent and continuous approach is needed.

A cluster randomised trial, FamilieTrivsel (‘Family Wellbeing’) [28, 29] is investigating the use of a web-based intervention focusing on parental mentalization skills and mental wellbeing and examines its impact on the child’s language and social development. The Family Wellbeing trial has implemented standardised child records with an enhanced focus on psychosocial aspects in both study arms with the aim of increasing the scope of the scheduled developmental assessments. This study takes a closer look at the clinicians’ experiences when using the standardised child records and investigates the clinical barriers and facilitators associated with the use of this approach to developmental surveillance.

## Method

### Aim

The aim of this study is to investigate clinical barriers and facilitators when introducing standardised child records with increased focus on psychosocial wellbeing and mental health into developmental assessments.

### Study design

This qualitative study is based on 12 interviews with GPs and midwives from general practice. Interviews were semi-structured to allow questions to be elaborated and participants to bring up topics. The research was nested in the Family Wellbeing trial mentioned above. Further details are reported elsewhere [28, 29]. Only control group clinics were included in this study to avoid bias from the web-intervention currently being examined in the Family Wellbeing trial.

The child records are structured and standardised templates to help clinicians incorporate important elements of the medical history and examination during scheduled developmental assessments at 5 weeks, 5, 12 and 24 months of age. The record templates were developed in collaboration with GPs [30] and had been tested in a pilot study with 10 clinics. Adjustments were made before implementation on a larger scale in the Family Wellbeing trial. In addition to the usual physical examinations, the standardised child records also contained topics related to socioeconomic factors, parents’ physical and mental wellbeing, information about siblings, relationships with grandparents and social network. It also provided a scheme for observing and recording the interaction between parent and child (‘parent–child interaction assessment’) and participating clinicians were taught to use this scheme to assess parent–child interaction. This was a simplified version of the Child–Adult Relationship Observation [8], which was found to be burdensome in its complete form during the piloting work. The simplified observational assessment encourages the clinician to observe dimensions of parent–child interaction that are known to be important for child development [8] but has not yet been validated. Three concepts are addressed in the parent–child interaction assessment: autonomy (respecting the child’s boundaries and viewing the child as an individual), responsiveness (understanding and responding appropriately to the child’s signals) and cooperation (preparing the child and collaborating). For each concept, it is noted whether it is possible to assess this during the consultation. If possible, it is noted whether the observation is positive or negative [8, 31]. Additional file 1 gives an example of a child record [32]. Prior to using the new standardised child records, GPs and some clinical staff (midwives and nurses), attended a one-day course where they were presented with the rationale behind the standardised child records and taught how to use them in practice.

### Setting

The study took place in Denmark, where GPs and/or their staff are responsible for three antenatal assessments and seven postnatal developmental assessments within a child’s first five years of life. Danish citizens have free access to healthcare and the standard of living is generally high. General practice handles around 80 percent of all initial patient contact, functioning as gatekeeper to the health care system. The antenatal and postnatal preventive programmes are managed by GPs in collaboration with hospital midwives (4–5 antenatal contacts) and municipality health nurses (5 visits during the child’s first year). The GP is, however, the clinician who has the most extensive contact with parents and children for the longest period of time [21]. General practices are run as profit-making businesses and each service generates a fee paid by the government. Consultations are free of cost to patients.

In Denmark GPs have a long tradition of writing short, informal notes in medical records. There is, however, an increasing trend towards GPs making templates to use in the daily consultations, hoping to save time while remembering to include important aspects.

Pregnant women were offered to participate in the Family Wellbeing trial consecutively at their first antenatal assessment if they understood Danish and if they intended to continue as patients in the practice [28, 29]. The present study took place in 12 general practices; three from Region Zealand and nine from the Capital Region. Clinicians saw a range of families with varied socioeconomic status. Clinics located in the most deprived areas were, however, less likely to participate in the Family Wellbeing trial and those that did had low rates of participant recruitment and record completion.

The Family Wellbeing trial received Regional ethics committee approval on 18^th^ October 2019 (KU approval no: 514–0362/19–3000).

### Data collection

Twenty-six clinics recruited families in the control group of the Family Wellbeing trial. One clinic only enrolled four families and was excluded as we decided that each clinic should include at least 10 families to gain sufficient experience with the child records. The remaining 25 clinics were invited to participate in interviews, and 16 clinics accepted. Variation in geography, municipality and gender of the GPs (clinic owners enrolled in the project) was prioritised when deciding the order of the interviews. Theoretical saturation was reached after 12 interviews since no new perspectives were emerging [33] and no interviews were conducted after that point. Participants gave informed consent to participate in the study and were reimbursed for their time. A topic guide (additional file 2) was designed by SV and GO. To cover most implementation-related aspects, the four generative mechanisms of Normalisation process theory (NPT) were considered when developing the topic guide [34, 35]. NPT is described under data analysis. Interviews were conducted by SV in June 2021 and subsequently transcribed verbatim.

### Characteristics of participants

Most GPs were female, and two of the three male GPs had female midwives carrying out their developmental assessments, leaving only one male GP to be interviewed. Seven of 12 clinics shared developmental assessments between the GP and a midwife. One clinic participated in the interview with both a GP and a midwife with equal experience in using the child records. The remaining clinics did not have the capacity for more than one clinician to attend the interview and only offered up the clinician with the most experience in using the child records. Of these, three interviews were conducted exclusively with midwives and the remaining eight interviews were exclusively with GPs. There was a wide variation in age and experience of the clinicians (Table 1).

**Table 1:** Characteristics of participating clinicians

**No**	**Sex**^a^	**Age** (years)	**Total experience** (years)	**Experience as GP** (years)	**Job**^b^	**Clinic**
1	F	60–69	20–29	20–29	GP	Solo (collab^c^)
2	M	40–49	10–19	0–9	GP	Partnership
3	F	30–39	10–19	0–9	GP	Partnership
4	F	60–69	30–39	20–29	GP	Solo (collab^c^)
4	F	40–49	10–19		MW	Solo (collab^c^)
5	F	50–59	30–39	20–29	GP	Partnership
6	F	50–59	20–29	10–19	GP	Partnership
7	F	40–49	10–19	0–9	GP	Partnership
8	F	50–59	20–29	20–29	GP	Solo (collab^c^)
9	F	30–39	0–9		MW	Solo^d^
10	F	40–49	0–9		MW	Partnership
11	F	40–49	10–19		MW	Partnership
12	F	40–49	10–19	0–9	GP	Partnership
**Mean**		**53**	**20**	**14**		

^a^*F* female, *M* male

^b^*GP* general practitioner, *MW* midwife

^c^*Collab* collaboration where the solo practitioners share staff and clinic facilities

^d^*Solo* practice with one owner but a large staff including several other doctors

### Data analysis

We initially coded and categorised data into factors that either promoted or inhibited professionals’ uptake of the child record [34]. Subsequently the coded data in these two categories were organised using the NPT framework [36, 35], which is a middle-range sociological theory that explains mechanisms promoting and inhibiting new practices becoming embedded in daily routine work across different contexts. The NPT framework encompasses four generative, explanatory mechanisms that affect the process of allowing a new practice to become integrated in clinical daily work. The mechanisms involve acceptance, engagement, integration and appraisal [36, 37]. During data analysis discussions were held regularly with all authors [34].

## Results

The findings are presented according to the four dimensions of NPT. Coherence describes how clinicians accepted the idea of increasing psychosocial focus in developmental assessments. Cognitive participation relates to how clinicians engaged with the standardised child records. Collective action describes mechanisms related to integration of the new practice and reflexive monitoring outlines how clinicians appraised the child records. Together, these dimensions shed light on barriers and facilitators when introducing the standardised child records into developmental assessments in general practice, in line with the aim of this study.

### Coherence: clinicians accepted the idea of increasing psychosocial focus in developmental assessments

#### Importance of developmental assessments

Developmental assessments formed a large part of the clinicians’ workload and they considered the preventive work an important part of their job. The clinicians found that antenatal and developmental assessments provided continuity of care, increased their knowledge of the families and strengthened clinician-parent relationship.

#### Interest in psychosocial aspects

Prior to the project, many clinicians were already interested in psychosocial aspects within the family. All clinicians were initially positive towards an increased psychosocial focus in the developmental assessments, and they participated in the study in the hope of becoming better clinicians and contributing to optimising the developmental assessments. A midwife reflected that the purpose of the developmental assessments has changed over the years, which is why the guidelines should be revised accordingly:

*“… you have not really revised the child health examinations since the 60s and the purpose of them, and back then there was high child mortality, and that is certainly not the case anymore. Then, how can we use these child health examinations?” – Midwife 9*

### Cognitive participation: it was novel for clinicians to ask families systematically about psychosocial factors

#### Relevance of the standardised child records

Most clinics had patients with high socioeconomic status and the pregnant women were generally well functioning. This led some clinicians to feel that increasing psychosocial focus was not relevant in their clinic after all and their engagement decreased over time. Others still found it important because families with high socioeconomic status could still have important psychosocial problems. Prior to the study some clinicians experienced that patients did not always share important psychosocial aspects of their lives on their own initiative:

*“... if the parents split up or something bigger happens, then it’s really important. And I have learned over the years that it is certainly not everyone who opens up about it.” – GP 8*

Therefore, some clinicians found that it made a lot of sense to approach these themes methodically, as they would during the somatic part of the examination.

#### Comparison to previous practice

A systematic approach to the developmental assessments was not new to clinicians. Many used self-made templates in the medical records, which were used as checklists during the consultations, so the concept of doing the assessment systematically integrated well with existing practice. Their systematic approach was, however, mainly applied to the physical examination prior to this project, and it varied how in depth the clinicians had recorded social conditions beforehand. Most clinicians found it novel to have the same kind of systematic approach when taking the medical history:

*“Something like social resources for example – it is new to ask about this. Relationships with grandparents is a new thing to ask about and then I have probably not asked so systematically about mental wellbeing.” – GP 6*

### Collective action: clinicians integrated the new practice in various ways

#### History taking

There were different opinions as to whether the systematic approach should be standardised. Some clinicians were confident that the parents themselves would bring up their problems during the consultation because the clinicians knew their patients very well, which also made many of the standardised question superfluous:

*“You don’t ask if grandparents are present (laughs), are your siblings there and all those family relations. I asked about that many years ago. I already know that.” – GP 1*

This led some clinicians to use only the parts of the standardised child records which they found to be relevant. Other clinicians went through all elements of the record every time as it ensured nothing to be overlooked:

*“I think it makes a lot of sense to go forward systematically, so you don’t forget or overlook anything.” – Midwife 10*

Some clinicians gained information that they believed parents would not have volunteered and thereby would not have been recorded without the standardised records.

*“Well, there is something about those family relations which has been made clearer, where I wouldn’t have caught it before [prior to using the child records]” – Midwife 11*

For instance some clinicians uncovered problems with sick grandparents early on and attributed this to the use of the standardised child record. In one of these cases having a sick grandparent contributed to the mother experiencing post-partum depression and the midwife considered it was helpful to know this early on. Applying the systematic approach of the standardised child records also became very useful in a case where the father was moving abroad. The parents were planning on sending the baby to family members in another country during that period. According to the GP all of this led to a planned visit to the home by the community health nurse, who discussed parent–child attachment with the mother and helped to get the child into a nearby day care service in order to keep him with his mother in Denmark.

Some clinicians found that parents would normally talk about certain subjects which they expected the clinician to be interested in. These would primarily be problems regarding the child and its physical wellbeing but less about the family and the child’s environment*:*

*“… they [the parents] only answer regarding how things are with the baby. So [shows tunnel vision with her hands] that is what you get, if you ask such an open question, you get answers to how the baby has slept last night or how much it has vomited… you very rarely get to know anything about how mom actually feels herself.” – GP 3*

Some clinicians found it awkward to ask questions about psychosocial aspects especially within resourceful families, and some clinicians completely neglected such questions if they had a feeling that there were no challenges at home:

*“These are some silly questions and it’s hard to ask them. We are located in an area where there are many well-functioning people. It’s not a socially burdened area.” – GP 5*

All clinicians believed parents should be able to influence what topics were discussed in the consultation and most desired some degree of free space during the consultations to avoid the conversation becoming too manualised. Some feared that using the child records could cause the conversation to be hasty and checking off boxes in a form could prevent parents from going into details and bringing up important elements spontaneously:

*“… having to ask, and then there will be a lot of no’s. It makes it a little easier to touch lightly on some things if there are a lot of no’s… it’s nice enough that it’s very specified, but… you may not focus on the one thing that matters the most.” – GP 2*

A few clinicians did not experience this dilemma as they used the child records as a conversation starter rather than a checklist, making it possible to have a loose conversation where these psychosocial aspects were included without the consultation becoming rigid. When the same questions were repeated at different developmental assessments clinicians found the record less workable and they feared that parents would find it tiresome.

#### Using the parent–child interaction assessment

Most clinicians had observed the interaction between parent and child to some extent prior to the study but the structured observation points in the record had facilitated a more conscious and systematic approach. While some found applying the concepts used to stimulate observation of parent–child interaction (“does the parent collaborate with the child?”, “is the parent sensitive to the child’s needs?”, “does the parent respect the child’s autonomy?”) *“really simple”,* others felt the concepts overlapped which made them difficult to note separately in the records. Nevertheless, the clinicians generally experienced being more aware of what to look for in the interaction between parent and child due to the use of the parent–child interaction assessment:

*“I actually think it has been really good, because it can be very easy to just say: “well, it’s all fine”, whereas here I have really noticed it.” – Midwife 10*

*“It actually supported me… It has become something I have implemented in my head when I observe a mother and a child.” – GP 8*

Moreover, there were examples of specific cases where the clinicians found the parent–child interaction assessment useful. One GP noticed a mother not carrying her child in a loving, caring way. Another GP experienced a case where the parents seemed to have problems understanding their child’s signals and responding properly. They started clapping over his head when he cried, which seemed to make him more distressed. A midwife described yet another case where a parent got frustrated during the developmental assessment:

*“When the little one starts to cry… she looks away and: “I just don’t know what to do, when he does that.” And then she can ignore him, and then the other one (parent) takes over” – Midwife 9*

Using the parent–child interaction assessment located problems sooner, thereby addressing them early on and helping the couples cope better.

#### Changes in work-flow

Some clinicians included items from the standardised child records in the medical history in every developmental assessment, where others incorporated the parent–child interaction assessment into their usual workflow. A few GPs planned to teach their colleagues about the parent–child interaction assessment, and some considered using the child record as a guide for trainee doctors.

Some clinicians changed their own behaviour during the developmental assessments. For example, one GP stayed more in the background – pretending to watch the computer for a while – thereby giving room for the parent to interact with the child. Other clinicians felt more professionally engaged in the developmental assessments compared to before. One GP emphasised that developmental assessments were no longer conducted as a physical checklist. All clinicians experienced some extent of increased focus on families’ psychosocial wellbeing*:*

*“… I think it has given a different presence – a different focus on wellbeing, both on mother and child – especially on the parents.” – GP 3*

### Reflective monitoring: clinicians gained increased psychosocial focus from using the standardised child records but they also experienced barriers when using them

#### Structured approach to developmental assessments

According to some clinicians the standardised child records with increased psychosocial focus provided an earlier detection of problems with wellbeing and gave a more nuanced and detailed picture of the family situation:

*“It was definitely an advantage to ask about their network and living conditions and all these things, which were actually specified.” – Midwife 9*

Many clinicians were ambivalent and felt some resistance towards the developmental assessments becoming too standardised:

*“I don’t think you can make anything standardised for every clinic. The old solo practitioner, who has to change the approach he had for the last 30 years, compared to a newly educated young female [doctor] who had just become a mother herself, I think they do things differently.” – GP 7*

#### Target groups

Several clinicians suggested that the child records should only be used with specific, vulnerable families or only in clinics located in more social disadvantaged areas. A midwife pointed out that it was beneficial to ask all families about psychosocial aspects, and to avoid parents from feeling stigmatised as particularly vulnerable she would say: “*I actually just have to ask about these things”.*

#### New vocabulary

The parent–child interaction assessment gave some clinicians a new vocabulary to describe what they observed, both in relation to documenting findings, but also if the parents needed guidance:

*“I could have used the words for what I thought was missing [e.g. in the parent child- relationship]. Sometimes you just can’t see what it is – where it is you don’t feel the parents hear or see the child. I have got words for that now. I just think those were the words I was missing back then to be able to describe it.” – GP 7*

#### Time frames

Most of the clinicians felt that more consultation time than the usual 15–20 min would be needed to go into detail about psychosocial wellbeing in the developmental assessments. Once an issue was brought up the clinicians did, however, find the setting of primary care useful because of the possibility to invite the families back for extended visits. When using the parent–child interaction assessment, some clinicians found it problematic that they only witnessed a short moment of interaction which could make the assessment difficult.

#### Sensitive topics

Some clinicians found it awkward to ask about personal matters – especially if it was not in line with parents’ expectations which revolved around physical aspects. Most clinicians felt it was difficult to comment on areas where parents could improve:

*“I think that can also be difficult, because it’s hard to criticise a mother who… does her best, right? And you have to be careful how you do it so that you still have their trust and they don’t feel like a bad mother, right?” – Midwife 11*

Discussing psychosocial aspects demanded that clinicians choose their words carefully in order not to push the parents away. Some clinicians hoped that showing interest in families’ psychosocial wellbeing would make it more *“straightforward”* for parents to reach out and seek help when needed.

#### Handling psychosocial issues

A few clinicians were frustrated by having to explore psychosocial issues more in depth as they felt they lacked options on how to act on concerns, and that they were unable to access services that could alleviate lack of social networks or parenting problems. On the other hand, some clinicians pointed out that just listening to the parents and letting them get something off their chest in a safe space could have a significant impact on its own.

*“… you’re also a pastoral carer when you’re a GP. You’re not just a doctor…” – GP 1*

Thereby emphasising that part of a GP’s job is to listen, give guidance and deal with emotional problems as well as physical and mental issues.

#### Legal concerns

Many clinicians thought that findings had to be of a specific character in order to document them because patients/parents have access to the record which could, potentially, be used in later litigation:

*“Well, you can say that a medical record follows you for your whole life and similarly in the context of insurance... It must be accurate, but you must be careful not to write anything that could harm the patient in the long run.” – GP 6*

In this project the record lends itself to writing about the parents in the child record, which raised another legal concern about third party information. One GP experienced a case where the father sought access to the children’s records in connection to divorcing the mother. According to the GP this could potentially have affected a family lawsuit if the record had said anything problematic about the mother’s behaviour or psyche.

## Discussion

### Summary of findings

Clinicians understood and valued the purpose of increasing the psychosocial focus in developmental assessments which they considered a useful and integrated part of developmental assessments. Facilitators included the systematic approach in history taking and assessment of parent–child interaction. The experience of benefits from using the child record also facilitated continuous use of the records. These benefits included increased knowledge of families, discovering psychosocial issues early on and having an increased focus and a larger vocabulary when observing and describing parent–child interaction. Barriers included that the history taking could become too rigid, leaving less room for the parents to bring up other issues. Furthermore, the use of a standardised approach also raised feelings of awkwardness and discomfort when clinicians felt forced to discuss sensitive matters and some felt they lacked solutions when parents disclosed delicate issues. Additional issues arose such as the need for extended consultation time and concerns when registering findings. Some clinicians thought standardised child records with psychosocial focus would be put to better use with especially vulnerable families or in more demographically challenged areas.

### Facilitators and barriers

*Facilitators:* using the standardised child records gave some clinicians extended information about the families and helped uncover important psychosocial issues that potentially needed to be addressed. Self-reported questionnaires regarding psychosocial aspects have previously proven useful during preventive child care visits as it led to the receipt of more community resources for families [38], which proves the benefits of a structured approach. Furthermore, health visitors have called for a more structured approach to assess parent–child interaction along with more training in this area [39]. Our study indicates that clinicians valued the structured approach from the parent–child interaction assessment of the child record including their new-found vocabulary. Since a shared language between health care professionals is associated with better inter-professional collaboration in healthcare teams and with higher quality of care [40, 41] the parent–child interaction assessment might have potential as an inter-professional communication-device. Simulation and use of standardised tools have demonstrated effectiveness in improving communication between healthcare professionals [42].

*Barriers:* in the present study some clinicians found the child records too rigid with their questionnaire-like structure and they wanted to be able to influence how the medical history taking was conducted. In line with this, it has previously been found that templates designed to ensure standards of quality of care in general practice contributed to bureaucratisation and may marginalise aspects of patient-centred care [43]. Furthermore, if the child records are used in a questionnaire-like manner the clinician might come across as insensitive or reserved, especially if psychosocial issues or concerns are not explored sufficiently. It has previously been found that an interested conversation style (as opposed to a reserved conversation style) facilitates the discussion of psychosocial aspects during developmental assessments [25]. Trust is an important component of the clinician-patient relationship and is needed in order to address sensitive topics [44, 45]. Some clinicians felt awkward asking sensitive questions, perhaps because they lacked communication skills or because the child records do not adjust to the fact that a relationship needs to be established before addressing such topics. In line with our findings, clinicians conducting preventive child health examinations want to improve their communication skills [39, 46]. Clinicians’ ability to listen and communicate is an important element in patient experience [44, 47], and trustful clinician-patient relationships along with positive responsiveness should enable parents to bring up psychosocial problems in the future. It has previously been identified that parental concerns mentioned during the developmental assessments were not subsequently explored and were often terminated by the clinician [48]. Moreover, psychosocial issues were repeatedly de-emphasised in favour of the physical examination [48]. This demonstrates that uncovering psychosocial aspects is not equivalent to handling psychosocial concerns properly. Results of our study also suggested that some clinicians felt a lack of options when handling psychosocial issues, which can lead to an unsatisfactory outcome of the consultation. Some clinicians found the child records superfluous as they knew their patients well or expected parents to disclose important psychosocial aspects by themselves. Doctors, however, do not know their patients’ and families’ vulnerabilities as well as they think [49, 50] and patients often do not bring personal matters up by themselves [51–53]. There exists a parental uncertainty about the purpose of the developmental assessments [48] which can also inhibit parents from bringing up psychosocial issues. Additionally, parental attitude has a great impact on the extent to which psychosocial aspects are explored [25] in line with our findings that clinicians find psychosocial aspects hard to address if parents are very physically focused. In addition, clinicians in our study had concerns about documenting psychosocial issues in the child record because it could potentially distress parents or children viewing the record: e.g. a mother reading she has inadequate interaction with her child or an older child reading about how the mother was depressed and distant towards him/her. These are nevertheless examples of important issues that need to be addressed and documented. The dilemma of how to help families in the best possible way without risking harm may lead clinicians to under-report observations that are cause for concern [54]. The record could potentially be used in insurance cases or legal proceedings and balancing the use of the record as a working tool but also potentially a legal document poses challenges. In general practice it might be possible to keep most information about family members separate, while it is more challenging in other settings where clinicians do not have access to parents’ records e.g. health nurse visits or paediatricians’ offices.

### Strengths and limitations

There were several strengths to this study. First, the sample includes a diverse group of clinicians: both GPs and midwives with a broad range of clinical experience. Second, the interviewer had no role in designing the child records which hopefully made clinicians less reluctant to be critical towards the records during the interviews. Third, the NPT strengthens the study as a proven theoretical framework for evaluating the implementation of innovation in the health service.

The study also had some limitations. First, the majority of clinicians who participated in the interview were female, perhaps lessening the diversity of views. Second, there are some potential biases attributable to sampling. The standardised records were made to be applied to the general population, but the clinics were generally located in demographic areas with relatively high-income families which potentially reduced the number of cases with psychosocial issues. Third, the clinicians who chose to participate in the Family Wellbeing trial might be more positive towards the idea of an increased psychosocial focus compared to other clinicians around the country. This could cause a challenge if an increased psychosocial focus scheme was implemented in other settings, as engagement is important in relation to the success of any implementation [36, 35].

### Implications for practice

Adjusting parental expectations to the developmental assessments, by shifting the mindset towards an increased psychosocial focus, might facilitate conversation about perceived stressors within the parents themselves, rather than exclusively discussing the child’s physical development. Converting parts of the child record into self-complete questionnaires filled out prior to the consultations, could contribute to an expectation alignment thereby making the consultation less rigid providing space for parents to bring up important issues. Barriers related to lack of time, resources, heavy workload and lack of financial incentives needs to be addressed for future implementation. Furthermore, clinicians who carry out developmental assessments need sufficient knowledge about coping strategies, referral options and training in communication techniques.

### Implications for research

First, parental attitudes towards an increased psychosocial focus in developmental assessments need further investigation. Second, more research is needed to demonstrate the impact of the parent–child interaction assessment on identification of problems. Third, the issue about what to write in a child’s record, what to give access to and whether the interests of parents or the child should be prioritised is an important dilemma which call for further research.

## Conclusion

A structured, standardised child record can be accepted and implemented in the context of developmental assessments in general practice. The record provides a universal and continuous approach necessary to assess risk factors and increase clinicians’ psychosocial focus on child wellbeing. An expectation alignment between clinicians and parents prior to the developmental assessments might be beneficial. Devoting more time and financial incentives to the developmental assessments could facilitate an increased psychosocial focus. Clinicians carrying out developmental assessments need, however, to be well-equipped in handling psychosocial problems through coping strategies, referral options and communication techniques. Brief assessment of parent–child-interaction bears obvious potential for identifying dysfunctional parenting behaviours and enhancing communication between health services and professionals and should be examined more thoroughly. Finally, further research is needed to explore parental views on benefits and disadvantages of developmental assessments with enhanced psychosocial focus.

## Supplementary Information

**Additional file 1.**

**Additional file 2.**

## Data Availability

The datasets generated and analysed during the current study are not publicly available because they contain confidential statements by participants about their patients. Edited/redacted transcripts of interviews can be made available by the corresponding author on reasonable request.

## Abbreviations

GP: General practitioner
GPs: General practitioners
