# Supplementary material for: Increasing the psychosocial focus in child developmental assessments: a qualitative study
Source: BMC Pediatr. 2023 Jan 25;23:44. doi: 10.1186/s12887-023-03849-x (PMC9875422; doi:10.1186/s12887-023-03849-x)
Supplement: Supplementary file 1 — Additional file 1. [file 12887_2023_3849_MOESM1_ESM.pdf]

Additional file 1

5 week preventive child health examination

Date: **Birth date:** **Examination by:** Doctor ☐ Midwife ☐ Nurse ☐

Mother's name: **Child's name:**

Who is accompanies the child:

The birth

Birth weight: ……………………… Born in week: ………………………

Birth complications: yes ☐ no ☐ Comment: ……………………………………………………

Neonatal complications: yes ☐ no ☐ Comment: ……………………………………………………

Conversation with the parents about the pregnancy, birth and new-born period: yes ☐ no ☐

Comment: ………

Family and social conditions

Parents living together: yes ☐ no ☐ Comment: ……………………………………………………

Parents healthy: yes ☐ no ☐ Comment: ……………………………………………………

Conversation about mental well-being: yes ☐ no ☐

The mother, problems: yes ☐ no ☐ Comment: ……………………………………………………

The father, problems: yes ☐ no ☐ Comment: ……………………………………………………

No. siblings: ….. , healthy yes ☐ no ☐ Comment: ……………………………………………………

Conversation about social conditions: (parents' jobs, economy, accommodation, social benefits) yes ☐ no ☐

Comment: ………

Relationships with grandparents: yes ☐ no ☐ Elaborate: ……………………………………………………

………

Conversation about network: (relation to siblings, family, friends, colleagues, neighbours) yes ☐ no ☐

Comment: ………

………

Current

Regulation of the child:

Problems with breastfeeding: yes ☐ no ☐ Comment: ……………………………………………………

Problems with sleep: yes ☐ no ☐ Comment: ……………………………………………………

Problems with circadian rhythm: yes ☐ no ☐ Comment: ……………………………………………………

Problems with crying: yes ☐ no ☐ Comment: ……………………………………………………

Illness/health issues since birth: yes ☐ no ☐ Comment: ……………………………………………………

Do the mother/parents experience problems in their care of the child?

yes ☐ no ☐ Comment: ……………………………………………………………………………………

Contact with the health nurse: yes ☐ no ☐ Comment: ……………………………………………

Health examination

Head measurement: ………….. Weight: ………….. Length: ………….. Weight gain pr. Week: …………..
(100-400 g +/- for both boys and girls)

Observation of collaboration, autonomy and responsiveness:

Mother-child:

Did you observe?	Yes /no	Positive	Negative
Cooperation			
Autonomy			
Responsiveness			

Comments: ………………………………………………

…………………………………………………………………

Father-child (or other companion):

Did you observe?	Yes /no	Positive	Negative
Cooperation			
Autonomy			
Responsiveness			

Comments: ………………………………………………

…………………………………………………………………

Was something worrying? ……………………………………………………………………………………………………

Physical examination:

Normal interaction with the child: yes ☐ no ☐ Comment: ……………………………………………
(The child listens and fixates its eyes briefly) ………………………………………………………

Overall normal assessment: yes ☐ no ☐ Comment: ……………………………………………
(Casual, comfortable with calm, symmetrical movements. Reflexes, tonus. Lifting the head from abdominal position. Crying with variation) ………………………………………………………

Normal eye pupils, red eye reflex: yes ☐ no ☐ Comment: ……………………………………………

Normal skull, mouth, skin, neck: yes ☐ no ☐ Comment: ……………………………………………

Normal heart/lung stethoscopy: yes ☐ no ☐ Comment: ……………………………………………

Normal hip joints: yes ☐ no ☐ Comment: ……………………………………………

Normal genitals: yes ☐ no ☐ Comment: ……………………………………………

Additional info: ……………………………………………………………………………………………………

Overall assessment

Short resume of important findings:

……

……

……

Follow up before next preventive child health examination:

Consultation-control ☐ Home visiting nurse ☐ Specialist/hospital ☐

Social services ☐ Other effort ☐ No extra effort needed ☐

Comment: ……………………………………………………………………………………………………

……
