# Supplementary material for: Increasing the psychosocial focus in child developmental assessments: a qualitative study
Source: BMC Pediatr. 2023 Jan 25;23:44. doi: 10.1186/s12887-023-03849-x (PMC9875422; doi:10.1186/s12887-023-03849-x)
Supplement: Supplementary file 2 — Additional file 2. [file 12887_2023_3849_MOESM2_ESM.pdf]

Additional file 2

Topic guide: Standardized child records used at developmental assessments in General Practice

Before the interview the doctor/nurse/midwife was asked to think of an easy and a difficult case of developmental assessments, where they used the standardized child records.

Background

- Would you like to tell me about you and this general practice?
- What was your motivation for participating in this project (Family Well-being trial)?
- What thoughts did you have on having a systematic approach in the record of the developmental assessment prior to this project?

Starting phase

- Try to describe how you remember the starting phase when working with the new, standardized child records.
    - What thoughts did you have on the new, child records prior using them?
    - When did you start using them?
    - How did you include patients?
    - What considerations did you do in terms of inclusion?
    - How well did you know the families you included?

Division of labour

- How is the labour divided between you and your colleagues?
    - Who does what during the child developmental assessments?
- Did you and your colleagues talk about, whether or not you use the child records in the same way?
    - If yes, can you elaborate?

Example with a 5 week record

The doctor/nurse/midwife is presented with a 5 week child record and asked if he/she wants to comment on it

- How did you use this?
- What do you like and dislike in the record?
- What is new to you and what did you already do prior to the study?

Using the standardized child records

- Which ones of the standardized child records do you have experience in using?
- How was your experience with using the new, standardized child records?
    - What came from using the child records?
    - Did you adjust anything to make the child records fit better into your daily work?
        - If yes, how?

  - What was different compared to the prior developmental assessments?
  - Did the use of the child records lead to any overall change in your behaviour at the child developmental assessments?
    - If yes, how?
  - Did using the child records have impact on your daily work?
    - If yes, how?
  - Did the new child record lead to changes in your standard phrases (templates)?
  - Did it lead to special conversations in the clinic?
    - If yes, how?
  - How much time did was reserved for the developmental assessments?
    - Was it fitting?

Psychosocial aspect in the standardized child record

- What is your experience with including psychosocial aspects in the developmental assessments compared to prior to the project (Family Well-being) trial?
  - Prior to the project, how did you include psychosocial well-being?
  - What is your opinion of being "forced" to focus on the psychosocial aspect?
  - Do you think a standardized child record could increase focus on mental health?
    - Why/why not?
  - What could argue for or against using the new, standardized child records?
  - How could the child records be designed in another way that still works across different professions?
  - How was it to observe "cooperation, autonomy and responsiveness" between parent and child?
  - How could the interaction be assessed in other ways?
- Did the parents themselves bring up psychosocial matters?

Potential challenges:

- What challenges have you faced when using the new, standardized child records?
  - Did you see anything worring in the parents or the child during the developmental assessments?
    - If yes, how did you handle it?
  - What difficulties are associated with assessing the relationship/interaction between parents and child?
  - Was it difficult or awkward to comment on the parent-child relationship/interaction?
    - If yes, how?
  - Are there problems related to registering you findings, where you are worried about the relationship/interaction between parents and the child?
    - Do you see any potential legal issues related to using the new, standardized child records?

Closing

- Do you have anything else you want to add about using the child records?
